# Gendered late working life trajectories, family history and welfare regimes: evidence from SHARELIFE

**DOI:** 10.1007/s10433-023-00752-3

**Published:** 2023-03-01

**Authors:** Wiebke Schmitz, L. Naegele, F. Frerichs, L. Ellwardt

**Affiliations:** 1 Federal Institute for Vocational Education and Training (BIBB), Bonn, Germany; 2 Department of Ageing and Work, Institute for Gerontology, University of Vechta, Vechta, Germany; 3 Institute of Sociology and Social Psychology, University of Cologne, Cologne, Germany; 4 Cologne Graduate School in Management Economics and Social Sciences, University of Cologne, Albertus-Magnus-Platz, 50923 Cologne, Germany

**Keywords:** Late working life, Family history, Gender inequality, Welfare regimes, Sequence analysis, SHARELIFE

## Abstract

Earlier employment choices based on family events in earlier life have an impact up until late working life, especially in welfare regimes that encourage the breadwinner-caretaker division. We investigate types of late employment patterns and how these are associated with earlier family events. We also test whether the association between early family history and late working life varies across five welfare regimes. Using retrospective life history data from SHARELIFE, our sample consists of 10,913 women and 10,614 men aged 65 years and older. Late working life trajectories are analyzed using gender-separate sequence analyses, which are summarized into eight groups applying cluster analyses. Using average marginal and interaction effects, we explain how the association between types of late working life, coresidential partnership history and parenthood history differs by welfare states. For instance, women’s late employment is either shaped by unpaid care or paid (full- or part-time) work but not both, whereas men’s late working life is mainly shaped by full-time work. Family history in earlier life is linked to unpaid care and part-time work—an association strongest in liberal and southern welfare regimes. However, among men earlier family events are linked to full-time work. Policymakers need gender-specific strategies to integrate workers into late working life. The implementation of new policies should aim to prevent these social inequalities in early life, as employment decisions caused by family history in earlier life stages—especially for women—tend to cumulate over the life course.

## Introduction

Aging societies have been challenged by a growing shortage of skilled workers and the rising costs of pensions (Lynch 2006). Consequently, policymakers have raised retirement ages to extend working lives and increase the labor market participation of older workers (Crossdale et al. 2022). However, this strategy deepens inequalities and puts disadvantages on those without opportunities to work longer (Mäcken et al. 2022; Bennett and Möhring 2015). Access to the labor market has remained impeded for certain populations, especially for women. This is unfortunate because the integration of women into the workforce is a particularly promising means of substantially enhancing the aging workforce. One explanation is that, compared to men, women are culturally expected to shoulder the lion share of unpaid care work (Meyer and Pfau-Effinger 2006). European countries largely rely on the family, and therefore women, to provide care and have not developed successful strategies to encourage women to remain in the labor force (Foster and Walker 2013). This is problematic because earlier employment choices (e.g. labor market exit, reduction of working hours) based on earlier family-related life course events such as childbirth, partnership, cohabitation, or divorce (hereinafter referred to as “family history”) have an impact up until late working life, which with variations across countries is understood as labor market participation beyond age 50 (Wahrendorf et al. 2018; Hoven et al. 2018). Consequently, women’s late working lives tend to be characterized by unpaid care work or part-time employment. In contrast, men’s late working histories turn out largely structured around full-time work (Komp-Leukkunen 2019; Stafford et al. 2019; Wahrendorf et al. 2018). The main purpose of this study is to, first, explore the working trajectories for the groups of men and women in late life, and second, associate these trajectories with family history across welfare regimes.

We add to and advance existing research in four ways. First, this is the first study to examine how the association between gendered late working life trajectories and earlier family history differs by five *welfare regimes*—including post-socialist countries. The majority of studies examined or compared single countries (Lacey et al. 2016; Ehrlich et al. 2020; Stafford et al. 2019; König 2017; Fasang 2010). However, the generalization of one country's findings has limitations because public policies vary across countries (Mayer 2004, 2009; Kuitto and Helmdag 2021; Möhring 2016). By comparing welfare regimes, we gain a better understanding of how individual life courses depend on different types of national contexts. Second, previous research has mostly focused on single outcomes and especially retirement timing to understand late employment (Madero-Cabib et al. 2015; König 2017; Fasang 2010; Toczek et al. 2022; Bennett and Möhring 2015). However, explaining retirement does not provide knowledge about those older people excluded from the workforce (e.g. women). Moreover, to understand late employment it is necessary to simultaneously inspect multiple indicators anchored in employment histories. Our sequence and cluster analysis contribute to closing this research gap by capturing trajectories of late employment over time. This allows us to use the actual late employment history of our sample as an outcome instead of single employment statuses (Aisenbrey and Fasang 2010). Third, much research has insufficiently addressed the explanatory role of early family history concerning late employment trajectories, also due to the focus on shorter time periods, such as the short-term effects of caregiving on employment (Bertogg et al. 2021; Lalive and Zweimüller 2009) or multi-channel work-family sequence analyses (Lacey et al. 2016; McMunn et al. 2015; Madero-Cabib and Fasang 2016). Few studies have employed a *life course perspective* to examine how late working life is associated with family history, such as childcare or coresidential partnership (Wahrendorf et al. 2018; Worts et al. 2016; Levy and Widmer 2013). A fourth shortcoming is the investigation of groups of men and women together when deriving employment history types (Wahrendorf et al. 2018; Hoven et al. 2018). This likely obscures meaningful differences between them because women’s employment histories are more disruptive than men’s (Komp-Leukkunen 2019).

This study tackles previous shortcomings by analyzing how the gender-specific association between family history and late working life histories differs across five welfare regimes. We use life history data from the Survey of Health, Ageing and Retirement in Europe (SHARELIFE) to answer the following research questions: Do working life trajectories in Europe differ by gender? Can these trajectories be explained by family history? Does the association between late working life trajectories and family history vary across welfare regimes? We carry out explorative sequence analyses for the groups of men and women 15 years prior to retirement (50–65 years). The resulting types of employment histories serve as the outcome in a multinomial regression framework with family history and welfare regimes as predictors.

## Theory and evidence

Previous research has suggested the suitability of the *life course perspective* in explaining working trajectories (Hoven et al. 2018; Madero-Cabib and Fasang 2016). Instead of examining static outcomes, the life course paradigm is dynamic, focusing on trajectories instead of single events (Aisenbrey and Fasang 2010) and depicts individual histories in changing and processual terms. Furthermore, individual lives are linked with those of others (“linked lives”), as people are embedded in relationships with people whose life experiences have consequences for them (Bengtson et al. 2016; Settersten 2015). For example regarding our focus on employment trajectories, people in a family or partner context might coordinate their work courses with one another in order to reconcile work and child care (Naegele and Walker 2021). Hence, life and work courses (in older age) are shaped, timed and ordered by inter- and intragenerational relationships as well as earlier events in life (Elder et al. 2003). Childbirth and divorce, for instance, have resounding effects on late working life, as they impact labor market participation and retirement timing, especially for women who still bear the majority of care work (Dingemans and Möhring 2019). Individual life courses are also influenced by socio-political frameworks as the reconciliation of care and work relies heavily on institutional settings and the availability of welfare (Dannefer 2003; Elder et al. 2003; Mayer 2004).

Moreover, according to the *theory of cumulative (dis)advantages*, adversities in earlier life accumulate into growing disadvantages which are enhanced through social characteristics (e.g. gender, access to education, class membership) (Dannefer 2003). In this article, disadvantages are generally understood as being excluded from the labor market because of the gendered burden of care. Women are more likely to have disruptive employment histories because of care responsibilities, which further enhances labor market exclusion risks and leads to involuntary retirement in late working life (Komp-Leukkunen 2019; Hoven et al. 2018). This holds especially true for women with lower socio-economic status (SES) (Brandt et al. 2022) which highlights the importance of applying an *intersectional perspective* when looking at cumulative disadvantages (Holman and Walker 2020). These pathways are exacerbated by additional risks. Individuals with low education and worse health status in earlier life are more likely to experience discontinuous employment histories or to exit employment permanently in later life (Hoven et al. 2018; Hyde and Dingemans 2017).

According to the *human capital theory*, individuals weigh costs and benefits when they choose between employment and unpaid work (Becker 1965). These choices are shaped by societal norms and their reproduction via policies (Dewilde 2003). If societal norms and associated welfare state policies assign care responsibility to women, remaining in employment will only be implementable with great hurdles (De Tavernier 2016). Hence, the resulting choices may be gendered because men and women have different opportunities presented to them and these decisions are assumed to impact employment up until late working life: For instance, past discontinuities in working life due to child-rearing among women cause less work experience which might decrease their chances of getting a job, which in turn further reduces their work experiences and employment chances over the life course. Previous research has shown long-term effects of earlier family events on late employment: Partnered women with children are more likely to be in unpaid care or part-time work in old age, whereas men are more likely to be employed full-time (Worts et al. 2016; Wahrendorf et al. 2018; Abendroth et al. 2014). However, societal norms such as traditional gender roles are assumed to be less prevalent among younger birth cohorts because of the modernization of gender arrangements where women are not necessarily expected to exit the labor force anymore to take care of family and because of women’s increasing attempt to combine employment and domestic work (Meyer and Pfau-Effinger 2006; Komp-Leukkunen 2019).

The welfare state is seen as an important factor shaping the structure of an individual’s life course. Their social security institutions and policies structure employment histories by rewarding continuous employment biographies (permanent full-time employment) which are mainly valid for men, whereas women are generally expected to follow normal family biographies (marriage, childcare) (Kohli 2007; Mayer 2004; Lewis 1992). Countries can be grouped into different types of welfare regimes based on three dimensions of welfare that impact individual employment histories: decommodification (the extent of key social security programs or interventions such as unemployment insurance, pensions, public childcare and sickness insurance), social stratification (the extent to which the welfare state increases or decreases levels of social stratification) and the mix of private–public family welfare (the role of the state, the family and the market in the delivery of welfare) (Esping-Andersen 1990). In the following we summarize and compare five types of welfare regimes regarding their impact on gendered work courses: (1) *social democratic*, (2) liberal, (3) *conservative*, (4) *southern* and (5) *post-socialist regime*.

Women’s attachment to the labor market is strongest in a (1) *social democratic regime* (e.g. Sweden), which supports flexible careers, the dual-earner model and public child care (Mayer 2004; Anttonen and Sipilä 1996). Countries of a (2) *liberal regime* (e.g. UK) support market mechanisms that produce welfare, which in turn encourage the traditional breadwinner-caretaker division. The (3) *conservative* (e.g. Germany) and (4) *southern regime* (e.g. Greece), on the other hand, rely heavily on women to shoulder care responsibilities (Worts et al. 2016): the *conservative regime* highly regulates working life by rewarding continuous working biographies, whereas the *southern regime* is characterized by a lack of intervention. Both regimes produce high levels of gender inequality (Möhring 2016). Labor market participation among women compared to men in later life is lowest in the conservative and southern regimes in contrast to the social democratic and liberal regime (Crossdale et al. 2022; Worts et al. 2016). Lastly, the (5) *post-socialist* regime (e.g. Czech Republic) is generally characterized by a high prevalence of female full-time employment and only short employment disruptions due to the provision of public child care (Buchholz et al. 2008; Möhring 2016).

Despite its popularity, Esping-Andersen’s typology has been criticized as limited and too simplistic: Welfare state orientations might change over time leading to countries needing to be re-allocated in the typology. Switzerland, albeit not being an undisputed case, has shifted toward the liberal model e.g., by strengthening the private sector in the delivery of welfare, while maintaining policies that are reminiscent of the conservative model (Arts and Gelissen 2002; Bonoli and Kato 2004; Obinger et al. 2010). Countries not only shift or combine characteristics of more than one welfare state, but they also differ within welfare regimes. This holds especially true in the case of eastern European countries, often simply grouped into the so-called post-socialist regime, which are not only very heterogeneous regarding their impact on employment careers (Möhring 2016; Komp-Leukkunen 2019) but have also experienced vastly different socio-economic developments since the collapse of the Soviet Union (Slukhai and Borshchenko 2019).

Based on these theoretical and empirical considerations, we argue that late working life histories differ heavily by gender. First, we hypothesize that women’s late working life is strongly shaped by unpaid care and part-time work compared to men, while men’s late working life is mainly characterized by full-time work (*H1*). Second, we expect that family history—and therefore the years in earlier life that respondents spend in a partnership and/or with children—contributes to these inequalities: We hypothesize that family history increases the women’s probability of being in domestic and part-time work (*H2a*), and the men’s probability of working full-time (*H2b*). Lastly, we hypothesize that the former associations between family history and late working life trajectories are most pronounced in welfare regimes that encourage the breadwinner-caretaker division (*H3*).

## Method and measurement

### Data and sample

We used retrospective life history data from the Survey of Health, Ageing and Retirement in Europe (SHARELIFE) (Börsch-Supan 2022; Börsch-Supan et al. 2013; Bergmann et al. 2019a, 2019b). Data collection took place in 28 countries in 2017, encompassing representative samples of individuals aged 50 years and over and their partners living in private households. Wave 7, which encompasses *n* = 77,261 observations, constitutes a retrospective survey covering employment, partnership and parenthood history among other topics. During the data preparation, we excluded observations with incomplete employment/job sequences (*n* = 1314; 2.22%), incomplete information on situations (e.g. domestic work, sick or disabled) between jobs (*n* = 1294; 2.18%), incomplete partnership (*n* = 1812; 2.87%) and parenthood histories (*n* = 1507; 2.38%) as well as cases with implausible information (e.g. if the reported year when respondents started a job is higher than the year when they left the job) (*n* = 1369; 2.31%). Moreover, because we intend to examine employment trajectories, only those respondents that had been in paid employment at least once in their life were considered in our analysis (5.93% of the full sample have never been employed). To obtain complete employment histories during the ages of 50 to 65 years, only those respondents aged 65 and older were included in our sample, which were born between 1912 and 1954. Furthermore, we eliminated observations of countries outside Europe and those that may not be categorized using the welfare regime typology (see below). Our sample consists of 21 countries summarized below. The final sample included *n* = 10,913 women and *n* = 10,614 men. As the liberal welfare regime, with only one country, (Switzerland) is underrepresented in SHARELIFE, we conducted additional robustness checks using data from the English Longitudinal Study of Ageing (ELSA) (*n* = 2120 women; *n* = 1699 men). The data from ELSA were collected in the UK in 2007 among a representative sample of people aged 50 + years (Banks J, Institute for Fiscal Studies et al. 2021). In doing so, we applied the same analytic procedure using ELSA to compare if the association between parenthood, partnership history and late employment trajectories among respondents in Switzerland is comparable to those in the UK. Because all relevant variables in ELSA are harmonized, they compare well with the measurements from SHARELIFE. However, we decided not to merge ELSA with SHARELIFE and therefore not to include UK in the main analysis because the timing of the data collection of both surveys is 10 years apart. ELSA therefore does not include information on respondents in younger cohorts compared to SHARELIFE. The results using ELSA are applied for robustness checks in order to support our results with SHARELIFE and can be viewed in appendix.

### Outcome

The employment module in SHARELIFE contains information on every job that respondents have had during their employment career for at least six months. Additionally, it yielded information on gaps during which respondents were not in paid work for six or more months. This information enables us to describe the late working life histories of individuals between the ages of 50 and 65 years. If there was an overlap between the year a respondent left a job and shifted to non-paid work, we coded that year as non-paid. The dependent variable *late working life* is measured using eight categories: (1) Full-time employed, (2) Full-time self-employed, (3) Part-time employed, (4) Part-time self-employed, (5) Domestic work, (6) Sick or disabled, (7) Unemployed or inactive and (8) Retired. Domestic work is interpreted as unpaid care work. However, wave 7 of SHARELIFE only includes the year of transitions from full- and part-time or vice versa between different jobs but not within the same job—we only know if respondents have answered having ‘changed multiple times between part- and full-time work’ in the same job. To solve this, we coded respondents as part-time workers if they have always been working part-time in the same job, changed once to part-time, or changed multiple times between full- and part-time in each job spell. Respondents are categorized as full-time employed if they have always been working full-time or changed once to full-time in this job.

### Predictors

The independent variable *parenthood history* counts the average number of adopted and natural children during the respondents’ ages of 25–49 years. While acknowledging that care responsibilities for women also include other family members (e.g. older relatives), we focus on childcare here, as we are interested in studying the effect of family events in earlier life phases. *Partnership history* regards the number of years respondents spent in a coresidential partnership during the age of 25–49 years. Respondents that lived at least 18.75 years (75% of 25 years) in a coresidential partnership are considered to have lived ‘mainly in a partnership’ (1) and are otherwise coded as ‘mainly without a partner’ (0).

### Moderator

To inspect variations across five *welfare regimes*, we categorized countries using a gender-sensitive typology (Komp-Leukkunen 2019). The social democratic welfare regime consists of Sweden, Denmark and Finland. The liberal regime includes only Switzerland. The conservative regime consists of Austria, Belgium, France, Germany and Luxembourg. The southern regime encompasses Greece, Italy, Spain, Portugal, Malta and Cyprus. The post-socialist regime contains Poland, Czech Republic, Hungary, Slovakia, Bulgaria and Croatia.

### Confounders

The analysis further controlled for *divorce*, which is coded 1 if respondents have ever been divorced in earlier life and 0 if not. Moreover we included *adult health*, measured by the number of periods of ill health or disability that have lasted for more than a year during adulthood. In addition, *child health* is subjectively rated on a scale from 1 (Excellent) to 5 (Poor), with higher values meaning poorer health during childhood. *Education* is coded into three categories using ISCED 97, which differentiates between primary or lower secondary education (1), secondary or post-secondary non-tertiary education (2) and bachelor, master or doctoral degree or equivalent (3). *Year of birth* was included as well. Prior research found that individuals who have been divorced, that have better health, higher educational levels and those in younger birth cohorts are more attached to the labor market in late life (Komp-Leukkunen 2019; Mäcken et al. 2022; Hoven et al. 2018; Dingemans and Möhring 2019).

### Analytical strategy

**Fig. 1:**
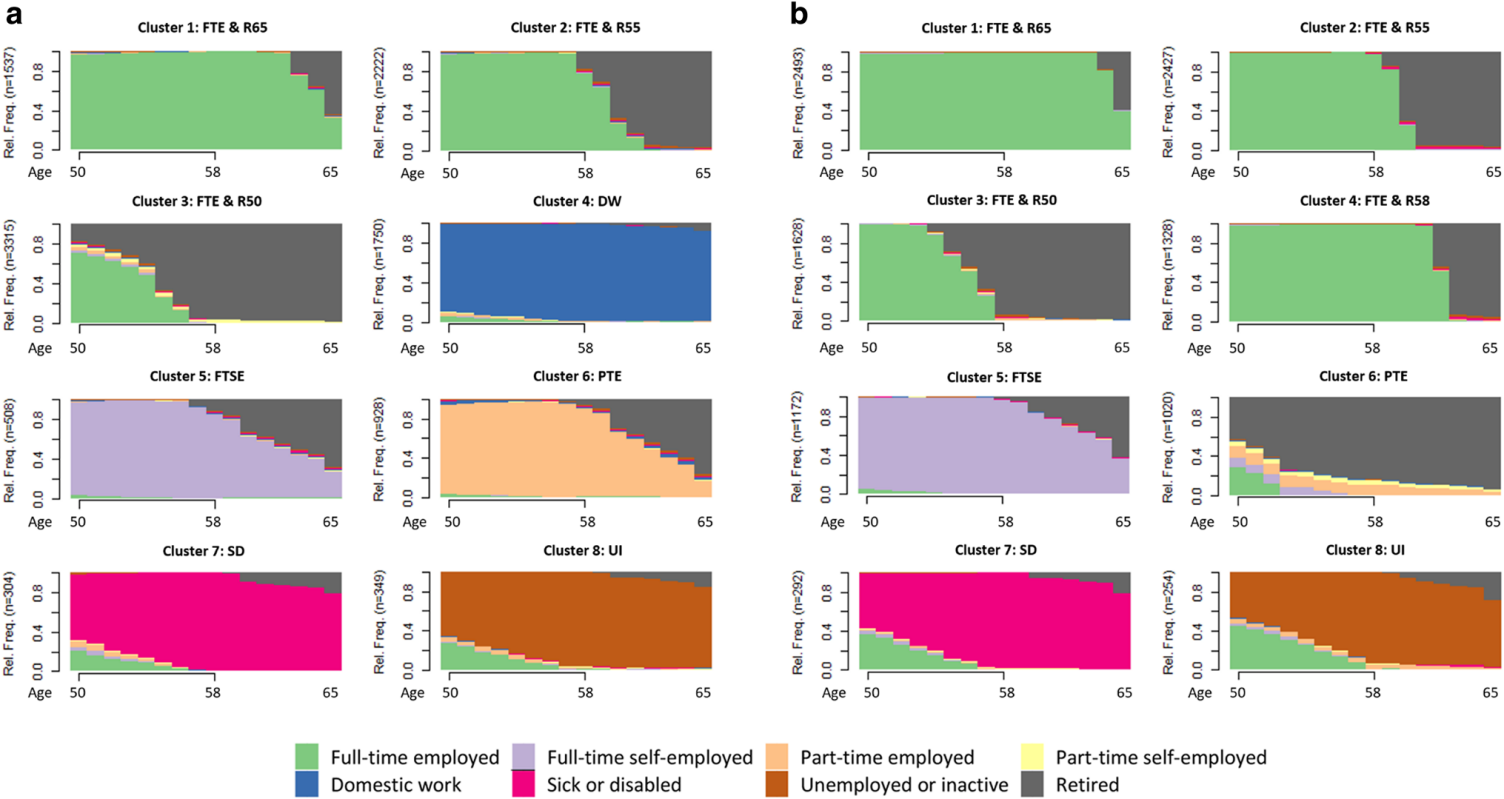
**a** **Women:** Late working life employment trajectories. Chronograms, *n* = 10,913. *Note*: ASW = 0.58; PBC = 0.67; HG = 0.89; Cluster labels: (1) FTE & R65: Full-time employed and retirement around age 65, (2) FTE & R55: Full-time employed and retirement around age 55, (3) FTE & R50: Full-time employed and retirement around age 50, (4) DW: Domestic work, (5) FTSE: Full-time self-employed, (6) PTE: Part-time employed, (7) SD: Sick or disabled, (8) UI: Unemployed or inactive; **b** **Men: **Late working life employment trajectories. Chronograms, *n* = 10,614. *Note*: ASW = 0.55; PBC = 0.52; HG = 0.87; Cluster labels: (1) FTE & R65: Full-time employed and retirement around age 65, (2) FTE & R55: Full-time employed and retirement around age 55, (3) FTE & R50: Full-time employed and retirement around age 50, (4) FTE & R58: Full-time employed and retirement around age 58, (5) FTSE: Full-time self-employed, (6) PTE: Part-time employed, (7) SD: Sick or disabled, (8) UI: Unemployed or inactive

Our statistical approach follows three steps: model individual trajectories, create a typology of trajectories and regress trajectory types on covariates. Specifically, in the first step, a *sequence analysis* models the late working life history of every respondent in the sample. In the second step, a *cluster analysis* explores distinct types of trajectories across respondents by comparing them using Optimal Matching (Studer and Ritschard 2016). Yet, while trajectories are highly homogeneous within clusters, trajectories are highly heterogeneous between clusters. This is achieved using Partitioning Around Medoids clustering (Studer 2013), as implemented in the *WeightedCluster* package in *R*, which uses matrices of pairwise distances. We compared solutions with lower and higher numbers of clusters. The best solution was chosen based on indicators for model fit, particularly the Average Silhouette Width (ASW), Point Biserial Correlation (PBC) and Hubert’s Gamma (HG) which are reported in Figs. 1a and 1b. Each cluster represents a subpopulation of respondents that follow similar trajectories. In the third step, we use *multinomial logistic regression* models to examine the association of late working life clusters with family history (partnership and parenthood history), and moderations thereof with welfare regimes. Results are expressed as average marginal effects (AME). The entire analysis is executed separately for the groups of men and women.

## Results

### Late life working trajectories

The sequence and cluster analyses revealed eight trajectories for both men and women. This solution indicated the best model fit, and its interpretation as well as cluster sizes were plausible. In both subpopulations, clusters were identified with an “Average Silhouette Width” (ASW) of > 0.5, which is considered a reasonable model fit (Studer 2013). Figure 1 displays all clusters using chronograms, where the horizontal line shows the prevalence of employment histories from the age of 50 to 65 and the ordinate represents the percentage of each occupational state for each age. Our *H1* hypothesized that women’s late working life is more frequently shaped by domestic and part-time work compared to men, while men’s late working life is mainly characterized by full-time work. Our findings support this expectation. However, our results show that women are either in paid work or domestic work in late working life, but there is no combination of both.

#### Women

Figure 1a shows that, overall, women’s late working life trajectories are diverse and thus mostly either structured around full-time, part-time or domestic work. They spent 4.56 years (SD = 5.34) in full-time employment, 1.42 years (SD = 3.84) in part-time employment and 3.24 years (SD = 6.20) in domestic work on average. Most women are in clusters 2 (20.4%) or 3 (30.4%), which are characterized by full-time work, and cluster 4 (16.0%), which is denoted by domestic work. Clusters 1, 2 and 3 are dominated by full-time work and only differ in the timing of retirement. Cluster 4 is almost completely characterized by domestic work and only a minority of women changed from full- or part-time work to domestic work. Part-time employed women are found in cluster 6, while full-time self-employed women are identified in cluster 5. Cluster 7 contains the lowest share of women (2.8%) and is dominated by those who have been working full-time and changed to the status of being sick or disabled. Most women in cluster 8 were unemployed or inactive and have previously worked in full- or part-time. Women have been sick or disabled 0.48 years (SD = 2.44) and unemployed or inactive 0.68 years (SD = 2.80) on average.

#### Men

Figure 1b shows that men’s late working life histories are less heterogeneous than women’s, with working biographies mainly structured around full-time employed and self-employed work. On average, men spent 8.08 years (SD = 5.58) in full-time employment and 1.91 years (SD = 4.68) in full-time self-employment. Most men are found in cluster 1 (23.5%) and cluster 2 (22.9%), which are dominated by full-time work. Cluster 1, 2, 3 and 4 are mostly characterized by full-time employment but vary by their timing of retirement. Cluster 5 is characterized by full-time self-employment and retirement around the age of 60 years. Cluster 6 can be described by transitions from full- to part-time until early retirement. Cluster 7 is dominated by men who changed from full-time work to being sick or disabled. Lastly, cluster 8 depicts the transitions of working full-time to being unemployed or inactive. On average, men have been 0.43 years (SD = 2.27) sick or disabled and 0.41 years (SD = 2.00) unemployed or inactive. However compared to women, they spent only 0.03 years (SD = 0.65) in domestic work and 0.21 years (SD = 1.59) in part-time employment on average.

### Distributions of trajectories by family history

#### Women

Table 1 presents the distribution of the previously identified clusters in Fig. 1a by coresidential partnership and parenthood history. Women who have mainly been without a partner and who have no children are frequently found in late working life trajectories characterized by full-time employment and later retirement. In contrast, single mothers, i.e., those without a partner and with children, are frequently found in full-time employment as well as the sick or disabled clusters. Their partnered childless counterparts often follow employment trajectories dominated by full-time employment, full-time self-employment and sickness or disablement. Lastly, women with a partner and children are typically found in domestic work and part-time employment.

**Table 1:** Women: Distribution of late working life clusters by coresidential partnership and children history (*n* = 10,913)

		Cluster									
		1 FTE & R65	2 FTE & R55	3 FTE & R50	4 DW	5 FTSE	6 PTE	7 SD	8 UI	Total	%
		%									
Mainly without coresidential partner	No children	12.4	8.9	5.0	1.2	4.0	2.0	6.4	2.7	543	5.0
	With children	12.5	11.6	9.6	6.7	4.5	7.9	13.2	8.9	1181	10.8
Mainly with coresidential partner	No children	5.9	5.6	5.6	3.9	5.7	3.9	8.3	4.4	600	5.5
	With children	69.1	74.0	79.8	88.2	85.8	86.2	72.0	84.0	8589	78.7
Total		1537	2222	3315	1750	508	928	304	349		
%		14.1	20.4	30.4	16.0	4.7	8.5	2.8	3.2		

Weighted; F = 6.71; *p* = 0.000; Cluster labels: (1) FTE & R65: Full-time employed and retirement around age 65, (2) FTE & R55: Full-time employed and retirement around age 55, (3) FTE & R50: Full-time employed and retirement around age 50, (4) DW: Domestic work, (5) FTSE: Full-time self-employed, (6) PTE: Part-time employed, (7) SD: Sick or disabled, (8) UI: Unemployed or inactive;

#### Men

Table 2 shows the distribution of the previously identified clusters in Fig. 1b. Overall, there was little variation in the prevalence of coresidential partnership and having children between clusters. However, taken together, full-time employment applied mostly to men with both a partner and children.

**Table 2:** Men: Distribution of late working life clusters by coresidential partnership and children history (*n* = 10,614)

		Cluster									
		1 FTE & R65	2 FTE & R55	3 FTE & R50	4 FTE & R58	5 FTSE	6 PTE	7 SD	8 UI	Total	%
		%									
Mainly without coresidential partner	No children	5.8	5.7	5.1	5.0	5.9	4.9	9.0	13.0	652	6.1
	With children	11.5	10.3	7.5	9.6	11.1	7.9	7.6	13.2	1156	10.9
Mainly with coresidential partner	No children	5.6	5.0	5.1	5.4	6.3	6.6	1.7	8.9	617	5.8
	With children	77.1	79.0	82.4	80.0	76.7	80.6	81.7	65.0	8189	77.2
Total		2493	2427	1628	1328	1172	1020	292	254		
%		23.5	22.9	15.3	12.5	11.1	9.6	2.8	2.4		

Weighted; F = 1.60; *p < .05; Cluster labels: (1) FTE & R65: Full-time employed and retirement around age 65, (2) FTE & R55: Full-time employed and retirement around age 55, (3) FTE & R50: Full-time employed and retirement around age 50, (4) FTE & R58: Full-time employed and retirement around age 58, (5) FTSE: Full-time self-employed, (6) PTE: Part-time employed, (7) SD: Sick or disabled, (8) UI: Unemployed or inactive

### Associations of trajectories with family history and welfare regimes

#### Women

Our *H2a* hypothesized that early family history increases the women’s probability of being in domestic and part-time work. The average marginal effects (AME) from the multinomial regression analysis shown in Table 3 largely confirm this expectation. In model 1, years of coresidential partnership and average number of children between the ages of 25 and 49 years were positively related to the probability of being in domestic work and part-time employment in late working life but negatively related to the probability of being in full-time employment. The number of children is also positively associated with sickness or disablement and full-time self-employment. In contrast, having spent more years in a coresidential partnership relates to a lower likelihood of being sick or disabled.

**Table 3:** Women: Average marginal effects (AME) based on multinomial regression analysis in percent (*n* = 10,913)

	Cluster															
	M1								M2							
	AME								AME							
	1 FTE & R65	2 FTE & R55	3 FTE & R50	4 DW	5 FTSE	6 PTE	7 SD	8 UI	1 FTE & R65	2 FTE & R55	3 FTE & R50	4 DW	5 FTSE	6 PTE	7 SD	8 UI
*Family history*																
Coresidential partnership	− 0.35***	− 0.51***	− 0.07	0.71***	0.03	0.25*	− 0.11**	0.06	− 0.35***	− 0.52***	− 0.07	0.70***	0.04	0.24**	− 0.10**	0.07
Number of children	− 1.64**	− 1.94***	− 0.44	2.01***	0.67***	1.04*	0.26*	0.03	− 1.61***	− 1.95***	− 0.47	2.12***	0.70***	0.93***	0.27**	0.01
*Regime (Ref. Social democratic)*																
Liberal	− 23.14***	− 15.92*	− 4.77	30.84***	2.70***	9.35	− 0.77	1.72**	− 22.97***	− 16.89**	− 4.90	31.11***	2.69***	9.78	− 0.94	2.12**
Conservative	− 25.07***	− 1.35	4.46	17.88***	2.90*	− 3.76	1.32*	3.63***	− 25.65***	− 1.35	4.61	17.88***	3.02*	− 3.55	1.29*	3.74***
Southern	− 18.81**	− 4.97	10.23	23.31***	4.57***	− 14.23*	− 0.53	0.43	− 19.73**	− 4.92	9.92	23.84***	4.97***	− 13.92*	− 0.60	0.44
Post-socialist	− 27.76***	− 0.72	43.57***	− 1.38	0.52	− 18.65**	4.83	− 0.41	− 28.32***	− 0.67	43.22***	− 1.14	0.94	− 18.33**	4.79	− 0.49
Regime* Coresidential partnership																
Social democratic	–	–	–	–	–	–	–	–	− 0.45**	0.02	− 0.23***	0.05	− 0.19*	0.80***	− 0.04	0.04
Liberal	–	–	–	–	–	–	–	–	− 0.21***	− 0.45***	− 0.16***	0.72***	− 0.20***	0.09***	− 0.02***	0.23***
Conservative	–	–	–	–	–	–	–	–	− 0.28**	− 0.78***	− 0.25*	0.94**	0.11	0.37**	− 0.07***	− 0.03
Southern	–	–	–	–	–	–	–	–	− 0.71***	− 0.49***	0.17	0.67	0.11	0.06	− 0.09***	0.28
Post-socialist	–	–	–	–	–	–	–	–	− 0.08	− 0.05	0.13	0.31**	− 0.14	− 0.04	− 0.17	0.05
Regime*Number of children																
Social democratic	–	–	–	–	–	–	–	–	− 2.55*	− 0.26	2.24**	0.49	0.29	− 0.48	0.29	− 0.02
Liberal	–	–	–	–	–	–	–	–	− 2.66***	− 2.46***	− 1.72***	4.15***	− 0.16**	2.38***	− 0.55***	1.02***
Conservative	–	–	–	–	–	–	–	–	− 1.92***	− 1.79***	0.29	2.22***	0.39	0.84***	0.13	− 0.16
Southern	–	–	–	–	–	–	–	–	− 0.27	− 1.12***	− 5.80***	3.81***	1.23***	2.03*	0.24***	− 0.12
Post-socialist	–	–	–	–	–	–	–	–	− 1.98	− 3.70**	3.67*	− 0.00	0.82**	0.06	0.67***	0.46***

Weighted; robust cluster estimator (countries); *p < .05; **p < .01; ***p < .001; Base category: Cluster 1; Controls: divorce, adulthood health, childhood health, education, year of birth; Cluster labels: (1) FTE & R65: Full-time employed and retirement around age 65, (2) FTE & R55: Full-time employed and retirement around age 55, (3) FTE & R50: Full-time employed and retirement around age 50, (4) DW: Domestic work, (5) FTSE: Full-time self-employed, (6) PTE: Part-time employed, (7) SD: Sick or disabled, (8) UI: Unemployed or inactive

**Fig. 2:**
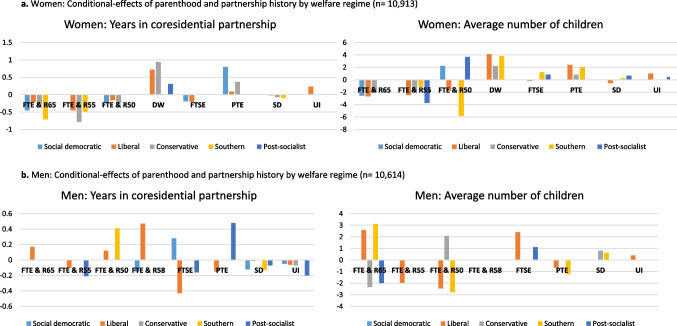
**a** **Women: **Conditional effects of parenthood and partnership history by welfare regime (*n* = 10,913). *Note*: Only significant results are reported; Controls: divorce, adulthood health, childhood health, education, year of birth; Cluster labels: (1) FTE & R65: Full-time employed and retirement around age 65, (2) FTE & R55: Full-time employed and retirement around age 55, (3) FTE & R50: Full-time employed and retirement around age 50, (4) DW: Domestic work, (5) FTSE: Full-time self-employed, (6) PTE: Part-time employed, (7) SD: Sick or disabled, (8) UI: Unemployed or inactive; **b** **Men: **Conditional effects of parenthood and partnership history by welfare regime (*n* = 10,614). *Note*: Only significant results are reported; Controls: divorce, adulthood health, childhood health, education, year of birth; Cluster labels: (1) FTE & R65: Full-time employed and retirement around age 65, (2) FTE & R55: Full-time employed and retirement around age 55, (3) FTE & R50: Full-time employed and retirement around age 50, (4) FTE & R58: Full-time employed and retirement around age 58, (5) FTSE: Full-time self-employed, (6) PTE: Part-time employed, (7) SD: Sick or disabled, (8) UI: Unemployed or inactive

Our *H3* hypothesized more pronounced associations between parenthood history, partnership history and late-working life trajectories in welfare regimes that leave women to shoulder care responsibilities. The results in Model 2, which includes the interaction effects of welfare regimes and family history, yield support for this hypothesis. All significant interaction effects are depicted in Fig. 2a. The associations between parenthood, coresidential partnership history and late-working life trajectories indeed vary by welfare regime and are strongest in the liberal, conservative and southern regimes. In the liberal, conservative and southern regime, parenthood history is positively associated with domestic work and part-time employment. Partnership history is positively related to domestic work in liberal, conservative and post-socialist regimes, whereas the association linking part-time employment and partnership history is strongest in social-democratic and conservative regimes. Having spent more years in a partnership in earlier life is negatively related to full-time employment in all regimes except the post-socialist regime. Those with a higher average number of children in earlier years are more likely in full-time employment in social democratic and post-socialist regimes and less likely in full-time employment especially in liberal, southern and conservative regimes. In the liberal and social democratic regime, women spending many years in a partnership are less likely to be full-time self-employed. Furthermore, women in the post-socialist and southern regime more likely to carry out full-time self-employment if they had many children.

#### Men

Table 4 reproduces the previous models for the population of men. Our *H2b* hypothesized that parenthood and partnership history increases the men’s probability of working full-time. Again, the findings appear to support this notion. According to model 1, men with long partnership histories more likely follow trajectories of full-time employment and less likely in sickness or disability and unemployment. Men with many children are more often found in sickness and disability.

**Table 4:** Men: Average marginal effects (AME) based on multinomial regression analysis in percent (*n* = 10,614)

	Cluster															
	M1								M2							
	AME								AME							
	1	2	3	4	5	6	7	8	1	2	3	4	5	6	7	8
	FTE & R65	FTE & R55	FTE & R50	FTE & R58	FTSE	PTE	SD	UI	FTE & R65	FTE & R55	FTE & R50	FTE & R58	FTSE	PTE	SD	UI
*Family history*																
Coresidential partnership	− 0.12	− 0.13	0.23**	0.01	− 0.10	0.30	− 0.10*	− 0.09***	− 0.15	− 0.15	0.25**	− 0.00	− 0.10	0.31*	− 0.06	− 0.09***
Number of children	− 0.52	0.23	0.26	− 0.41	0.38	− 0.46	0.60*	− 0.08	− 0.44	0.38	0.11	− 0.34	0.34	− 0.47**	0.53**	− 0.10
*Regime (Ref. Social democratic)*																
Liberal	0.14	1.77	− 2.40	− 1.09	6.17***	− 4.49***	0.59**	− 0.70	0.45	1.55	− 2.67	− 0.79	6.09***	− 4.57***	0.61***	− 0.66
Conservative	− 21.69**	7.74	10.57	− 3.03	3.50**	1.45	0.69***	0.75	− 21.81**	7.69	10.66	− 3.02	3.44**	1.53	0.78***	0.72
Southern	− 11.44	− 0.72	5.70	− 4.64***	5.56	6.09*	− 0.38	− 0.18	− 11.46	− 0.84	5.74	− 4.67***	5.42	6.26*	− 0.31	− 0.14
Post-socialist	− 18.29*	6.73	5.91	− 3.22	− 3.96	6.11**	5.68*	1.04	− 18.28*	6.74	6.02	− 3.29	− 4.15*	5.93**	6.08*	0.95
Regime*Coresidential partnership																
Social democratic	–	–	–	–	–	–	–	–	0.24	− 0.01	− 0.14	− 0.15***	0.28*	− 0.05	− 0.12***	− 0.05***
Liberal	–	–	–	–	–	–	–	–	0.17***	− 0.10**	0.12***	0.47***	− 0.43***	− 0.15***	− 0.01*	− 0.06***
Conservative	–	–	–	–	–	–	–	–	− 0.12	− 0.07	0.27	− 0.03	− 0.15	0.11	0.05	− 0.07***
Southern	–	–	–	–	–	–	–	–	− 0.38	− 0.25	0.41***	− 0.05	− 0.08	0.55	− 0.13***	− 0.08
Post-socialist	–	–	–	–	–	–	–	–	0.00	− 0.21*	− 0.03	0.20	− 0.16*	0.48**	− 0.07*	− 0.20***
Regime*Number of children																
Social democratic	–	–	–	–	–	–	–	–	− 0.90	1.55	0.38	− 0.61	1.22	− 0.89	0.41	− 1.17
Liberal	–	–	–	–	–	–	–	–	2.57***	− 1.97***	− 2.45***	− 0.22	2.41***	− 0.66***	− 0.07	0.38***
Conservative	–	–	–	–	–	–	–	–	− 2.34***	− 0.20	2.07*	− 0.39	0.78	− 0.15	0.80***	− 0.57
Southern	–	–	–	–	–	–	–	–	3.09***	0.89	− 2.78**	− 0.02	− 0.85	− 1.23***	0.64***	0.25
Post-socialist	–	–	–	–	–	–	–	–	− 1.99*	0.30	0.99	− 0.09	1.11**	0.28	− 0.29	0.54

Weighted; robust cluster estimator (countries); *p < .05; **p < .01; ***p < .001; Base category: Cluster 1; Controls: divorce, adulthood health, childhood health, education, year of birth; Cluster labels: (1) FTE & R65: Full-time employed and retirement around age 65, (2) FTE & R55: Full-time employed and retirement around age 55, (3) FTE & R50: Full-time employed and retirement around age 50, (4) FTE & R58: Full-time employed and retirement around age 58, (5) FTSE: Full-time self-employed, (6) PTE: Part-time employed, (7) SD: Sick or disabled, (8) UI: Unemployed or inactive;

In line with our *H3*, these associations are particularly pronounced in welfare regimes that encourage the breadwinner-caretaker division. In model 2, especially in the liberal and the southern regime, men are more often engaged in full-time employment when they have spent many years in a coresidential partnership and have had a higher number of children. In the conservative regime, men with many children are more likely to be sick or disabled. All significant interaction effects are shown in Fig. 2b.

## Discussion

This study employed a life course perspective to explore gender-specific late working life trajectories, and to explain them in relation to earlier family history and how they vary by welfare regimes. Gaining knowledge on how late working life patterns and its determinants differ for women and men, we aim to understand gender gaps in labor-market participation rates and how these gaps might be amplified or narrowed by welfare state orientations.

Using retrospective data from SHARELIFE and gender-separate sequence analyses, we found evidence that late working life histories differ dramatically by gender. In line with previous research (Komp-Leukkunen 2019), women’s late employment histories are either characterized by paid (part- or full-time) work or domestic work, while men’s late working life is mainly shaped by full-time work. This suggests that women decided between either paid or unpaid work, whereas the continuous normal employment biography mainly applies to men in our cohorts (Kohli 2007). However, prior research indicates that women in younger cohorts, which are not yet included in the SHARELIFE sample, are more successful at switching between paid and unpaid care work and therefore increase their labor market participation. A possible explanation is that cultural perceptions of gender arrangements as well as institutions (e.g. Discrimination Act at workplaces in Sweden) are changing (McMunn et al. 2015; Crossdale et al. 2022).

Moreover, in our study, late working life trajectories were associated with earlier family events in different ways among men and women. The average number of children over the years and years in a coresidential partnership were positively associated with unpaid domestic work or part-time work but negatively associated with full-time employment in later life in women. The same family events were not related or even inversely related to employment among men—i.e., there was a greater chance of full-time employment for partnered men. This supports the notion of the breadwinner-caretaker division showing that family events are more strongly related to women compared to men up until late working life—especially in southern and conservative welfare regimes (Killewald and García-Manglano 2016; Wahrendorf et al. 2018; Worts et al. 2016).

### Theoretical implications

Our results underscore arguments from the *cumulative disadvantage theory* (Dannefer 2003). Early family events—such as childbirth and therefore care responsibilities—may be carried through life until older age and prevent women from following typical male employment trajectories in old age (Kohli 2007). Because these labor market disadvantages due to the care burden pertain almost exclusively to women, family events appear to be gender-specific, which stresses arguments from the literature on gendered life courses (Levy and Widmer 2013; Moen 2001; Holman and Walker 2020). Our findings indicate that women who had more care responsibilities and have spent more years in a coresidential partnership in earlier life may have decided to exit from the workforce or to work in part-time to balance work and care responsibilities, and continued this employment pattern up until late working life. We assume that women might have difficulties to follow continuous full-time employment trajectories once they have chosen to exit from the labor force due to care responsibilities in earlier life—in contrast to men. This also mirrors the *linked lives* approach, assuming that women and men within a partnership coordinate their work courses with each other to reconcile care and paid work: women shoulder care work, whereas men take over the role of the breadwinner (Bengtson et al. 2016; Naegele and Walker 2021).

Moreover, our results support the *human capital theory* (Becker 1965) by showing that the impact of family history differs by welfare regimes, suggesting that women’s opportunities are dependent on the national context such as policies or societal norms (Möhring 2016; Fortin 2005; De Tavernier 2016). The previously discussed gender inequalities were particularly visible in *southern regimes*—characterized by a lack of public social infrastructure—and *conservative regimes*—which support continuous full-time employment as a standard for men but not necessarily for women. There was no such association in the *social democratic regime*, where women’s late employment is almost unaffected by family history. This is likely the result of social-democratic policies that support flexible employment by combining paid work and childcare (Kuitto and Helmdag 2021). This mirrors previous results showing higher female labor market participation in northern Europe (Anttonen and Sipilä 1996; Anxo et al. 2006). However, our results regarding *post-socialist regimes* turned out to be mixed. An explanation might be that the countries in this regime differ in their degree of female labor market integration as a result of different social policy reforms (Möhring 2016): For example, as the only post-socialist country, Poland significantly restricted the role of the state in providing social welfare and instead assigned the responsibility to individuals and their families—and therefore to women to shoulder family care (Steinhilber 2006).

**Fig. 3:**
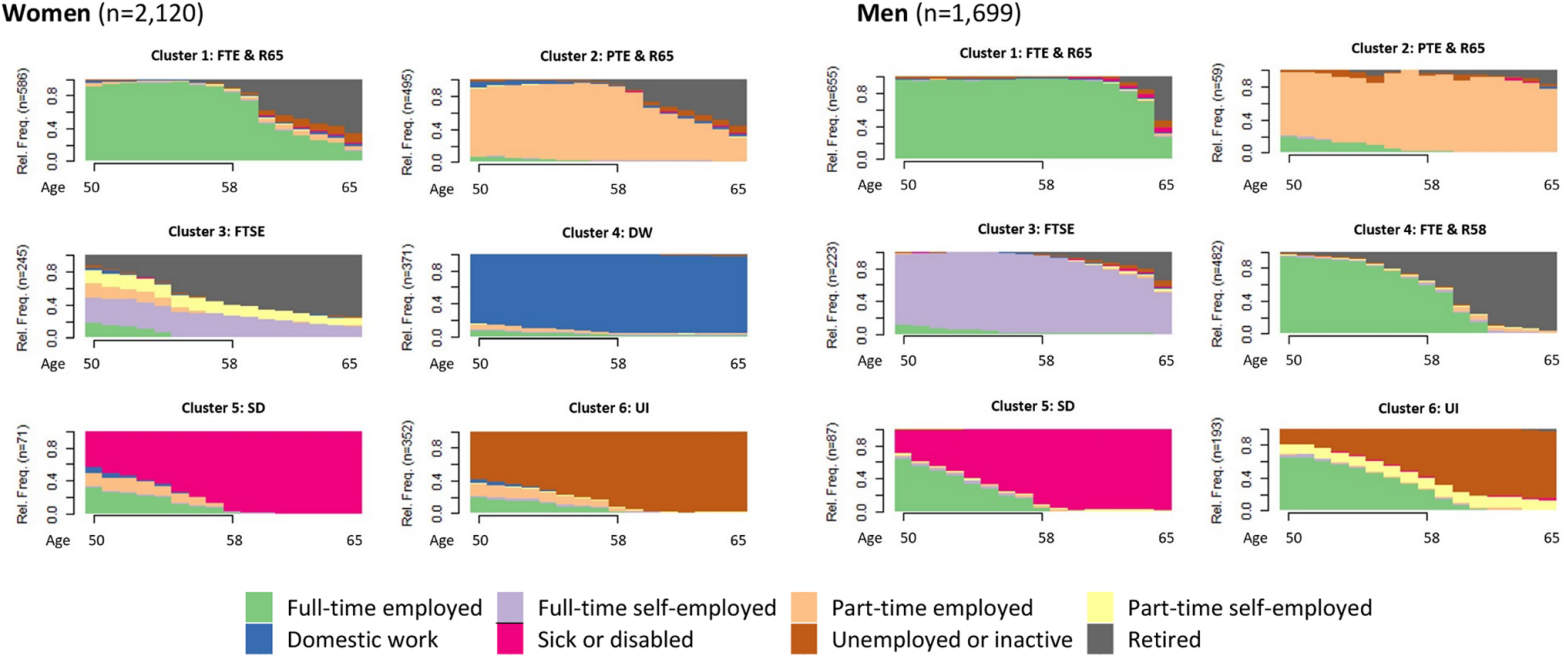
Robustness checks using ELSA: Late working life employment trajectories; Chronograms. **Women**: *Note*: ASW = 0.57; PBC = 0.81; HG = 0.94; Cluster labels: (1) FTE & R65: Full-time employment and retirement around 65, (2) PTE & R65: Part-time employment and retirement around 65, (3) FTSE: Full-time self-employment, (4) DW: Domestic work, (5) SD: Sick or disabled, (6) UI: Unemployed or inactive. **Men**: *Note*: ASW = 0.52; PBC = 0.66; HG = 0.84; Cluster labels: (1) FTE & R65: Full-time employment and retirement around 65, (2) PTE & R65: Part-time employment and retirement around 65, (3) FTSE: Full-time self-employment, (4)FTE & R58: Full-time employment and retirement around 58, (5) SD: Sick or disabled, (6) UI: Unemployed or inactive

Specifically, a strong association pertained to the *liberal regime*, represented by Switzerland. This finding connects well to previous evidence on the Swiss case, showing that women with a family history are more likely to be in long-term caring or unemployment (Madero-Cabib 2015; Madero-Cabib and Fasang 2016). An explanation for this finding is that Swiss policies tend to neglect workers that disrupt their employment career to look after family. We suggest that the Swiss case may be generalized to other liberal regimes. To assess the robustness of our findings, we compared the results from Switzerland with those from another liberal regime, namely the UK (Wahrendorf et al. 2018): A post hoc analysis of the representative survey ELSA produced very similar results (Appendix Table 5, Fig. 3). We therefore conclude that, especially in liberal regimes, mothers tend to pursue a career in part-time employment or domestic work, while fathers continue working full-time.

**Table 5:** Average marginal effects (AME) based on multinomial regression analysis in percent

	Women (*n* = 2120)						Men (*n* = 1699)					
	Cluster											
	AME						AME					
	1	2	3	4	5	6	1	2	3	4	5	6
	FTE & R65	PTE & R65	FTSE	DW	SD	UI	FTE & R65	PTE & R65	FTSE	FTE & R58	SD	UI
Coresidential partnership	− 0.91***	0.66***	− 0.15	0.35*	− 0.16**	0.22	− 0.09	− 0.04	0.12	0.53**	− 0.20**	− 0.32**
Number of children	− 0.97	0.55	− 1.3*	1.95**	0.87**	− 1.08	0.66	− 0.10	0.22	− 2.68*	0.34	1.57*

**Women:** Cluster labels: (1) FTE & R65: Full-time employment and retirement around 65, (2) PTE & R65: Part-time employment and retirement around 65, (3) FTSE: Full-time self-employment, (4) DW: Domestic work, (5) SD: Sick or disabled, (6) UI: Unemployed or inactive. **Men:** Cluster labels: (1) FTE & R65: Full-time employment and retirement around 65, (2) PTE & R65: Part-time employment and retirement around 65, (3) FTSE: Full-time self-employment, (4)FTE & R58: Full-time employment and retirement around 58, (5) SD: Sick or disabled, (6) UI: Unemployed or inactive

Weighted; *p < .05; **p < .01; ***p < .001; Base category: Cluster 1

Besides, we find that both men and women with a higher average number of children over the years in earlier life are more likely sick or disabled. Previous studies found that having children is linked to less wealth in later life (Plotnick 2009) which in turn has been found to decrease health, for example because of less economic security and not being able to afford a healthier lifestyle (Pollack et al. 2007). Another possible explanation for this finding is that mothers who spent most of their earlier years as housewives instead of in paid work, lack health promoting opportunities such as social interaction, fulfillment at work or financial independence: Prior research has shown that women who spend most of their life in unpaid care work—compared to women who were mostly full-time employed—were more likely affected by disability and mortality in later life (Benson et al. 2017; Sabbath et al. 2015; Lahelma et al. 2002).

Another interesting finding is that women with more children in earlier life have a higher probability of being in full-time self-employment in later life. This reflects previous research which found that women with more family responsibilities chose to be self-employed. Self-employment might allow them to be more self-determined, flexible and therefore to better balance work and family care demands (Wellington 2006; Joona 2017).

Lastly, we found that women who have been divorced in earlier life and have a higher educational level, are more likely to be in full-time employment and less likely to be in domestic work in late working life. This reflects previous research (Dingemans and Möhring 2019; Mäcken et al. 2022) and indicates, with regard to divorce, that women who lost their partner are forced to provide for themselves. Women with a higher educational level, on the other hand, might have a higher income and are therefore not dependent on their partner’s income compared to lower educated women.

### Limitations and suggestions for future research

Our study has several limitations. First, we measured childcare only indirectly through proxy indicators of parenthood history by assuming that the demand for care work increases with the number of children. Third, we observed spells of jobs and gaps between jobs that are at least 6 + months long, which might underestimate the complexity of late working life trajectories. Fourth, the data did not contain information concerning the mechanisms between family history and late employment: Family events might impact late working life through the loss of labor market expertise. Fifth, our findings cannot be completely generalized to younger cohorts that are starting a family nowadays—particularly because the traditional gendered division of paid and unpaid work is less strongly pronounced in younger generations (Meyer and Pfau-Effinger 2006). However, our results are still relevant for younger cohorts by showing the importance of national policy regimes for gendered opportunities to work longer. This is especially so because none of the countries included in this study have implemented any life course-oriented strategies yet to support women’s employment throughout their life. Lastly, the use of welfare typologies blurs differences across countries: Family policies may fit more than one regime type. Yet, further partitioning the data by country would have yielded small samples and low statistical power for our analyses. Moreover, factors such as cultural values and characteristics of the labor markets should be considered because employment is not just influenced by welfare regimes. More research is needed to better understand the gender-specific role of national contexts across the life course, including cultural norms and social policies, as well as the mechanisms linking family history and late employment.

## Data Availability

The data from ELSA were made available through the U.K. Data Archive. ELSA was developed by a team of researchers based at the NatCen Social Research, University College London and the Institute for Fiscal Studies. The data were collected by NatCen Social Research.

## Appendix

See Tables 1, 2, 3, 4 and 5.
