# Correction: Gendered late working life trajectories, family history and welfare regimes: evidence from SHARELIFE

**DOI:** 10.1007/s10433-023-00756-z

**Published:** 2023-04-10

**Authors:** Wiebke Schmitz, L. Naegele, F. Frerichs, L. Ellwardt

**Affiliations:** 1 Federal Institute for Vocational Education and Training (BIBB), Bonn, Germany; 2 Department of Ageing and Work, Institute for Gerontology, University of Vechta, Vechta, Germany; 3 Institute of Sociology and Social Psychology, University of Cologne, Cologne, Germany; 4 Cologne Graduate School in Management Economics and Social Sciences, University of Cologne, Albertus-Magnus-Platz, 50923 Cologne, Germany

**Correction to: European Journal of Ageing (2023) 20:5** 10.1007/s10433-023-00752-3

Following publication of the original article [1], the authors would like to correct the Funding information. The sentence should read:

Open Access funding enabled and organized by Projekt DEAL. Our study is part of the research project EIWO “Exclusion and Inequality in Late Working Life”. This work has been funded by the Swedish Research Council for Health, Working Life and Welfare (FORTE)—2019–01,245 & the Deutsche Forschungsgemeinschaft (DFG, German Research Foundation)—454,899,823.

The original article has been corrected.
